# The psychosocial beliefs, experiences and expectations of children living with obesity

**DOI:** 10.1111/hex.13973

**Published:** 2024-01-18

**Authors:** Lisa Newson, Nicola Sides, Amineh Rashidi

**Affiliations:** ^1^ School of Psychology, Faculty of Health Liverpool John Moores University Liverpool UK; ^2^ Ofcom London UK; ^3^ School of Nursing and Midwifery Edith Cowan University Joondalup Australia

**Keywords:** childhood, obesity, paediatric, psychological, well‐being, qualitative, weight management

## Abstract

**Background:**

Childhood obesity has been shown to impair psychological health. However, psychological factors are often overlooked in both research evaluations and treatment interventions, and children's perspectives on managing obesity are underexplored. Neglecting psychosocial factors might undermine interventions. This research explored the psychological beliefs, expectations and experiences of children living with obesity (range 7–13) and attending a weight management programme (WMP).

**Methods:**

Thirty‐four participants (19 females, 15 males, average age 9.5 years) completed a semistructured interview. Recorded interviews were transcribed verbatim and analysed using thematic analysis.

**Results:**

Four overarching themes were developed: (1) defining health and self‐recognition; (2) external influence; feedback, stigma and comparison; (3) recognising emotions and (4) future expectations: obesity is a reality. These themes interact to influence the children's psychosocial status.

**Conclusions:**

This study highlights a range of psychosocial and emotional difficulties that children living with obesity experience and suggests that these remain regardless of their attendance at a WMP. Interventions for children living with obesity should address psychosocial factors, including stress management, peer victimisation and handling feedback from others.

**Patient or Public Contribution:**

As proposed by the two young people acting as patient and public involvement and engagement representatives, the utilisation of scrapbooks as a preinterview tool was particularly helpful in aiding discussion during the interviews. This innovative approach could be considered a valuable methodological technique for investigating sensitive topics with children in future research.

## 1 INTRODUCTION

Childhood obesity is linked to various physical health issues,^1^ significantly impacting both morbidity and mortality.^2^, ^3^ Additionally, it is associated with adverse psychological health.^4^, ^5^, ^6^

Children living with obesity are more likely to engage in bullying or face bullying at school and experience social discrimination,^7^ leading to an increased risk of depression, relationship issues and behavioural challenges.^8^ Compared to their normal‐weight peers, they are more likely to experience low self‐esteem,^9^ depressive symptoms during adolescence^10^ and reduced quality of life, similar to children diagnosed with cancer.^11^, ^12^ Moreover, the longer a child is overweight, the higher the risk for mental health disorders.^13^ Childhood obesity has been associated with increased experiences of mental health diagnosis including anxiety, depression and experiences of suicidal thoughts, body dissatisfaction and hopelessness.^14^, ^15^

The majority of childhood obesity treatment interventions are conducted by multidisciplinary teams, offering multicomponent behavioural treatment.^16^ These interventions typically include diet, physical activity (PA) and behavioural components, but their success rates vary across studies.^16^, ^17^, ^18^, ^19^ A review^16^ analysing 217 childhood obesity interventions found that most studies focused on reporting weight change as the primary outcome (various metrics were used to assess relative adiposity, including body mass index [BMI], BMI standard deviation score, absolute weight, BMI percentile, percentage over median BMI and other measures). Despite offering a behavioural treatment intervention, only half (48%) of the studies reported behavioural outcomes, such as assessing changes to moderate‐to‐vigorous PA, reductions in television viewing and improvements in dietary intake (e.g., reduced fast food and calorie intake, increased consumption of fibre, fruits and vegetables). Noteworthy, however, that only 20% of the studies systematically reported psychosocial outcomes, with the most common being quality of life, and only 5% of the studies recorded mental health outcomes (such as depression).^16^ Despite the relationship between obesity and psychological variables and the increased risk of psychological comorbidities in individuals living with obesity,^20^ there is a lack of attention given to psychological variables as part of obesity treatment,^21^ especially within UK guidelines.^22^, ^23^, ^24^ This lack of focus on psychological variables is partly due to insufficient research on interventions that systematically assess psychological factors.

In 2011, the National Obesity Observatory report on obesity and mental health in the UK stated that ‘there is an urgent need for evaluation of interventions, both in terms of the weight loss and psychological benefits’.^25^ However, more than a decade later, the prevalence of childhood obesity continues to increase. In England, by the time children reach eleven years old, over a third (37.8%) are already categorised as being overweight or obese.^26^ The United Kingdom has one of the highest rates of childhood obesity among European countries, although similar statistics are reported across other countries^27^, ^28^ and worldwide.^29^

Despite this continual increase in children living with obesity, ‘little is known about the psychological effects on children of increasing their awareness of their condition of obesity’.^16^, ^30^ In summary, there is a lack of information on how or if interventions address the psychological needs of children living with obesity. This research explored the psychological beliefs, expectations and experiences of children living with obesity and attending a weight management programme (WMP). The research objectives were to examine the psychosocial issues children experience and highlight ways in which services may improve to meet the needs of these children.

## 2 METHODS

### 2.1 Design

Qualitative methodology was utilised to explore children's reality of attending a community WMP. We considered their experiences of being defined as living with obesity and the meanings attached to them. An inductive data‐driven reflexive thematic analysis (TA) has been applied to this study as it provides a theoretically flexible approach capable of providing detailed accounts and exploring patterned meaning across the whole data set.^31^, ^32^ TA has been used extensively across health and well‐being research and is particularly relevant to applied research settings, such as weight management.^33^ The WMPs were commissioned by Public Health‐Local Authorities and conducted in community settings. Therefore, Liverpool John Moores University, United Kingdom, approved the research ethics and completed a full independent peer review and monitoring process.

### 2.2 Participants

Fifty‐nine children living with obesity (BMI > 95th percentile) and who attended one of three independent community WMPs during 2018, were eligible and invited to participate in this research study. The WMPs, although independent of each other, adopted a range of behavioural change techniques^34^ to improve the children's lifestyles. They typically addressed eating, shopping, PA and sedentary behaviour by promoting self‐monitoring, providing instruction and demonstrating new behaviours. The programmes were family‐orientated and delivered in community venues after school. Staff on these programmes were from dietetic, leisure or sports services and reported receiving training in motivational interviewing. The interventions did not claim specifically to evaluate or treat specific aspects of psychological distress or the child's well‐being. However, many of these programmes aimed to teach the children to make sustainable lifestyle changes and thus improve the quality of life for these children. The programme outcomes focused on slowly reducing BMI percentiles over time, and secondary outcomes aimed to improve eating, PA behaviours and quality of life and well‐being. The programmes are deemed as being broadly representative of community WMPs. According to the health profiles for the areas, WMPs 1 and 2 were located within areas of high deprivation, whereas WMP 3 had lower‐than‐average deprivation.^35^

To establish a relationship with participants and build rapport,^36^ the first author attended the first two sessions of each WMP as a volunteer and was introduced to the families as a researcher and psychologist. These sessions helped build rapport and familiarisation. Conversations during these introductory sessions were not considered part of the research data collection. Forty‐one families were introduced to the first author during this time and were subsequently provided with information about this research study and initial consent to take part. As part of the research study preinterview process, the children were given a scrapbook which contained blank paper and some pre‐selected exercises.

#### 2.2.1 Patient and public involvement and engagement (PPIE): The scrapbook

PPIE was conducted with two young people in preparation for the study materials and interview process. The scrapbook idea was developed in consultation with these young people who had previously attended a WMP and considered themselves living with obesity. The purpose of the scrapbook was to help the children think about the interview topics in advance to help them process their thoughts before the interviews.^37^ As children were invited onto the study, they were given a scrapbook with pre‐populated questions/activities from the interview schedule and told: ‘before we do an interview, please look in the scrapbook; if you want to do the suggested activities that would be great, or if you want to write anything down to talk about when we meet you can do so. If you want to use the scrapbook but not share the contents that's also okay. If you are happy to bring the scrapbook to the interview, we can talk about what you have put in it’. The children had these scrapbooks for a minimum of 4 weeks before the interview.

#### 2.2.2 Interviews

Interviews were arranged from Week 6 of the intervention onwards at a convenient time for the children and conducted in the family home or at a community venue. Written parental consent and verbal child assent were sought before the interviews, which included consent for recording the interview using selected quotations in the analysis. If the participant offered extracts from their scrapbooks, we also copied these to use any written data in the analysis. The children did not have to answer questions or could stop the interview anytime. One‐to‐one interviews were deemed most appropriate as the children were asked to talk about their feelings, opinions and experiences of obesity.^38^

Thirty‐four children (see Table 1) agreed to complete interviews, 19 females and 15 males; mean age 9.5 years old (range 7–13). Of these, 29 brought along the scrapbook to the interview. The scrapbooks were scanned, and content was copied for 21 of the children (5/29 children brought along the scrapbook which had nothing in, and 3/29 had added to the scrapbook but asked for it to be kept private and not shared as part of the research interview). Of the 21 children who had used the scrapbook and were happy for this to be used, 17 referred directly to the scrapbook to help them express their feelings during the interview. The children were able to opt out at any stage of the study.

**Table 1:** Participant characteristics.

Participant number	Gender—male (m)/female (f)	Age (years)	Weight management programme attended	Parent/guardian present during interviews
1	m	8	1	Yes
2	m	7	1	Yes
3	f	9	1	
4	m	12	1	
5	f	10	1	
6	f	11	2	
7	f	7	1	Yes
8	m	8	2	Yes
9	m	11	2	Yes
10	f	10	1	
11	m	10	2	
12	m	7	2	Yes
13	m	9	1	
14	m	10	2	
15	f	8	3	Yes
16	f	7	3	
17	f	9	3	
18	f	10	2	
19	m	12	1	
20	m	13	3	
21	f	12	2	Yes
22	f	11	3	
23	f	8	1	
24	f	9	1	
25	f	8	2	Yes
26	f	11	3	
27	f	13	1	
28	f	9	1	
29	f	11	3	
30	m	8	2	
31	m	10	2	
32	m	10	2	
33	m	8	2	
34	f	7	2	Yes

### 2.3 Interview procedure

To offer a reassuring and supportive environment, the children were asked if they would like their parent or another person with them during the interview. Four children had their parents with them, and six asked if their parents could be in the room but not next to them. The interview was intended to be open and exploratory, led by the participants, and not influenced by a preconceived theoretical view of the interviewer. All participants were presented with the same initial questions (Table 2). Depending on their responses, further questions were asked to help them expand on their answers.

**Table 2:** Example interview questions.

▪ How was that scrapbook? Did you make any notes or drawings in the scrapbook which you want to tell me about? Would you show me? Did you draw in the scrapbook?—What did the past, present and future picture look like? (Its ok if you didn't use the scrapbook/Its ok if you don't want to show me/share).
▪ What does the word healthy mean to you?
▪ Why did you come on this (WMP insert), what was it for and how do you feel about it?
▪ Tell me about coming to this WMP *(name of the programme*) what happens.
▪ How does this programme make you feel? What has been good or not so good about this WMP?
▪ How would you describe the WMP to your friends?
▪ If we waved a magic want and tomorrow you woke up and things were different, what would that look like, how would you know? (What would it be like for you?)

Abbreviation: WMP, weight management programme.

### 2.4 Research team

The first author conducted the digitally recorded semistructured interviews. She was a registered health psychologist and a qualitative researcher. As a practitioner psychologist, she had experience providing NHS clinical support regarding weight management to children and families. She was sensitive to the needs of the children during the interviews, although she was not involved with the intervention linked to any children recruited into this study. Additionally, every fourth interview prompted discussions among the authors about the interview process and content and initiated the initial analytical reflections. At the time of this research, the second author was a weight management practitioner on a national programme, although not linked to the interventions investigated in this study. The third author, a chronic diseases nurse, experienced promoting lifestyle and changing behaviour.

### 2.5 Data analysis

The length of interviews averaged 37 min (range 6–74 min). One child (Participant 21) provided 6 min of interview and then stated she was bored and wanted to watch her favourite TV programme; therefore, the interview ended, though she also provided written text (answering all prequestions with additional comments and drawings) within her scrapbook. The interview was included in the analysis due to her unique and thoughtful insights spoken during the interview and the relevant feedback written in her scrapbook, as deemed valid by the research team. Another interview (Participant 25) ended early at 29 min due to the parent having other commitments. All other interviews came to a natural ending, and participants were debriefed and encouraged to reflect on their research experience. The digital recordings were transcribed verbatim, and the second author checked transcripts against recordings for accuracy. Written notes from the scrapbooks were appended to each participant's transcripts. All personally identifiable material (e.g., names) were replaced with alternatives.

Data included both the verbatim transcript and notes made from the scrapbooks, and therefore data was considered within a contextualist approach, allowing the flexibility of combining multiple sources of data for each participant.^31^, ^32^, ^39^ The data were subjected to reflexive TA,^31^ and the analysis adopted an inductive approach, working with the data from the bottom‐up.^40^ This involved exploring the participants’ perspectives within the context of attending the WMP. The analysis aimed to identify patterns within and across the data, seeking to construct a narrative that captures the experiences of the children. We applied reflexive TA,^31^, ^39^ incorporating its six phases in an iterative manner as follows: First, the authors read and reread the transcripts to become familiar with the breadth and depth of data being discussed; next, data were entered into NVivo.^41^ Initial codes were generated, by the lead researcher, across the whole data set relevant to the research question. Codes and the attached data were then arranged into potential themes, and the relationships between codes, sub‐themes and themes were considered and reworked. Subsequent creation and discussion of themes occurred through conversations between the authors, ensuring those themes were applied to the related coded extracts and the data set. We, the authors, recognise our active role in the analytical process and interpretation of the data.^42^, ^43^ Care was taken to reflect on the analytical process throughout. Our analytical strategy was inductive and data‐driven, identifying and discussing the salient themes repeated across and within transcripts. Themes were reworked and subsequently validated across the data. Finally, a thematic map was generated, themes were defined, and transcript quotations were selected to illustrate the themes identified. Within the quotes chosen … represents a pause in speech, and the authors have added words in brackets to aid readability. At the end of each quote, the participant's identifiers are provided: for example (P6f, 11 yrs, WMP2) refers to participant number 6, gender (female or male), age, WMP 1, 2 or 3. The raw data supporting this study's findings are available as quotations within the article; examples of text from the scrapbooks have been included where appropriate. Due to the nature of this research, in line with legal and ethical processes, participants did not agree for their full transcripts to be shared publicly, so supporting data beyond the sample data extracts is not feasible.

## 3 RESULTS

**Figure 1:**
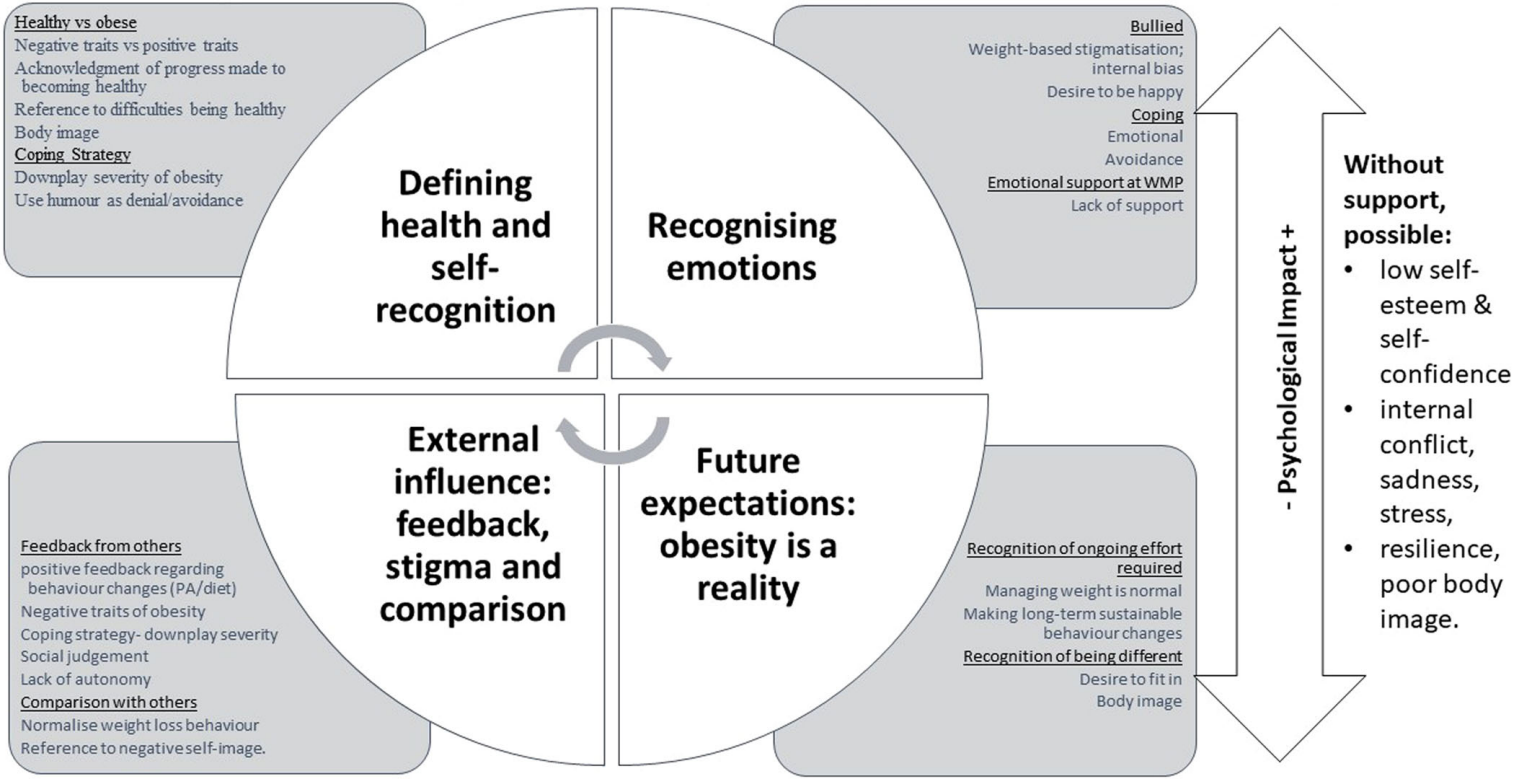
Thematic representation. The psychological experiences of children living with obesity.

Four themes were developed: Defining health and self‐recognition; external influence—feedback, stigma and comparison; recognising emotions and future expectations—obesity is a reality (see Figure 1). These themes were not isolated concepts but overlapped and influenced each other. Together they influenced the psychosocial world of the children.

### 3.1 Defining health and self‐recognition

The first theme explored how children conceptualised obesity. Drawing comparisons between what it means to be healthy or obese, while exploring their coping mechanisms. Children generally portrayed a negative image of obesity, contrasting it with an idealistic image of health associated with physical attractiveness and the absence of disease:

To be healthy is looking fresh, fit, you know what I mean, attractive, so you would say. Your body works perfectly; you can run a marathon proper fit. Your skin glows, and your hair is shiny; you don't take Paracetamol every day or get on the scales (P6f, 11 yrs, WMP2).

The children were able to acknowledge the behavioural progress they had made to become healthier:

I'm getting better, I'm eating fruit and stuff now not as many jellies (jelly sweets), and I do like like like doin' games, and jujitsu is a good one. (P7f, 7 yrs, WMP3)

However, nearly all the children reported practical barriers in their ability to become healthy.

I like strawberries, and melon, and raspberries, but I can just go to the shop and buy chocolate which I love love love it's so easy, but I have to go to the supermarket to get the fruits, and my pocket money isn't that good. (P22f, 11 yrs, WMP3)

Barriers were also related to their perceived ability to be good at something:

I'm not very fit; I don't dos very good at PE (physical education class), I like chips, but I am trying a bit. I could do a bit more, don't tell my mum I said that. (P1m, 8 yrs, WMP1)

And

nice people gets me doing things. Sometimes I just want to be good at something. I am very good at Minecraft (computer game), so if people do it with me, then I do it. (P18f, 10 yrs, WMP2)

While these physical and behavioural barriers were presented, they also represent the children's internal (high) expectations and the children's acknowledgement of wanting ‘to be good’. The children often described obesity as a negative trait, associating it with a less appealing physical appearance:

being overweight, is lazy, not the best looking, not fit. (P28f, 9 yrs, WMP1)

Humour and downplaying the severity of their situation presented as coping mechanisms, revealing the children's awareness and management of their obesity status:

You can die of fat, you know. It stops your heart working and makes your liver fatty, and it makes your joints ache. I need to nip this in the butt, ha‐ha, right now before it's too late and I explode ha. (P19m, 12 yrs, WMP1)

Internal conflicts were managed through a mix of humour and minimising the perceived severity of their situation, showcasing the complexity of how children navigate and define their experiences with obesity. In the quote which follows, the child is referring to his weight percentile chart and feedback from his father:

I'm only a little bit on the chart, not really (overweight), not a big deal. My dad says I don't need to come here, so I probably am quite healthy. (P31m, 10 yrs, WMP1)

### 3.2 External influence; feedback, stigma and comparison

Children's perceptions of their obesity status were significantly influenced by external feedback, particularly from family and friends. While some parents downplayed the reasons for attending WMPs:

My mum just says don't worry, we will sort it, but to be honest, I'm not sure what that means. (P12m, 7 yrs, WMP2)

children were generally aware of their purpose, linking it to healthy eating and trying new activities:

Mum says I'm here to learn about eating healthily and to try out new activities. I say I'm here because I have a wobbly tummy. (P18f, 10 yrs, WMP2)

Some children were able to process feedback from family members:

granny thinking that it really good I'm comin' here, and taking ‘responsibility’ for what am eatin' and not playing on the computer. (P13m, 9 yrs, WMP1)

Mixed communication from family members created anxiety for some children, who expressed a desire to please everyone:

I'm here because said so. I'm just trying to please everyone. My dad won't come here. He not liking me come here, so I can be a tinsey (tiny) bit sad. (P30m, 8 yrs, WMP2).

And

I come here cause I have to. Yes, I do like some of the stuff, but OMG (oh my god), sometimes I just want to stay at home. My mum makes me come, and I hate her, not really, but arrrhhh, so makes me. (P20m, 13 yrs, WMP3)

The concept of receiving safe feedback from family and friends was acceptable, but it was important for the children to ‘fit in’ and not be different from their peers. The children were very aware of the negative social judgement they may receive if other people knew they were attending:

Some of my friends know bout this that I come here, and so, yep, they are good. But sometimes, people don't know, and I don't want them to… I don't want them to think I'm, you know, I'm not nice. (P18f, 10 yrs, WMP2)

Positive feedback was acknowledged to increase their confidence and self‐worth:

what I like about it here is that *name*, is really nice and encourages me even when I don't want to do something, cause I think I'm a bit rubbish at it, but then she says come on, let's try and I do and its great, so now I know I can do it. (P30m, 8 yrs, WMP2)

and

My teacher told me the other day that I'm doing well, that's a first, and she put me in the first team for netball. I was well pleased. (P6f, 11 yrs, WMP2)

These comments demonstrate how the children's self‐esteem and self‐confidence are fragile. In addition to feedback from others, children made direct comparisons to significant others around them. The older children (10 yrs+) were able to normalise weight loss behaviour in adults:

Why not? Everybody does this sort of thing at one time or another, errm like right, going to Weight Watchers, Slimmer's World, I think this should be a bit more like that, then I know I will be losing weight each week. (P18f, 10 yrs, WMP2)

Comparison to those trying to lose weight were expressed with negative language:

Why do I need to come here? Well, I think we all know the answer to that. I am hashtag F. A. T., and I need to do something about it. Otherwise, I will end up like her (a reference to mother) … ‘oh, she's always on a diet, complaining she don't look good in stuff, always aching. (P10f, 10 yrs, WMP1).

The impact of external influences on children's self‐perception and attitudes towards weight management reflects the complexity of their experiences within the context of family, peers and societal norms.

### 3.3 Recognising emotions

Many of the children reported negative feelings due to living with obesity. The children described experiencing some form of bullying (verbal or physical) as a result of their obesity:

My nickname is Chubbs; it means fatty, right? thing is, even when I get thin, my nickname might still be fatty chubb chubbs, and then I'm old, right, like thirty‐something, maybe I will be still be called it, and if not, I will think back to when I was (called it)… I don't mind it sometimes cause everyone knows me, but I would like a nicer name, like Griff; he's the cool kid gets all the best. (P19, male, 12 yrs, WMP1)

The children described avoidance and emotional coping techniques to deal with peer victimisation and the realisation of their obesity:

I get angry, no, not sure of the word when I don't know what to do, I get a bit cross, so I sometimes stay away from the others, and sometimes I try not to think about it and join in, I hope that I get picked (for the team at school sports). (P4m, 12 yrs, WMP1)

It is noteworthy that some of the children did not believe that their psychological well‐being was being directly addressed while attending the WMP:

I thought this was going to sort me out, but not really. It's fun, but I still get bullied at school, I still cry sometimes … psss don't tell my mum I said that, okay? Yes, they do games and things on food and they tell us how to do things, but I just want to feel happy, and sometimes when I gets on my own, I'm not. (P14m, 10 yrs, WMP2)

Children acknowledged that the WMPs were helping them to improve their lifestyles, and it helped build their confidence in trying new activities and foods:

this (WMP) helps me eat more fruit, do assault course. I like talking to people about my things, but but most people don't have the time to make me feel good about things though, or let's be honest, it can be a bit too much, and then they overdo it. (P27f, 13 yrs, WMP1)

They reported that the WMP provided a safe and motivating environment for them to try new activities. However, they reported that this confidence and motivation was not present in their daily lives:

Okey‐dokey, so yes, I'm happy to do the games and play sports, you know, not really sports but the exercises while I'm at ‘programme name’, but defo not when I'm at school and no way would I do an afterschool club, I would look hashtag horrendous. (P10f, 10 yrs, WMP1)

Again, the children acknowledge how the WMP encouraged them to talk about how they felt about trying new foods and activities, although they thought that the programmes did not help them deal with their emotions:

We don't talk about feelings on their own; we just talk about how I feel about doing activity, that's different. (P28f, 9 yrs, WMP1)

### 3.4 Embracing the future: Obesity is a reality

Some of the very youngest children (aged 7) held idealistic views about their future, focusing on their body image:

I will be lots thinner and happy'. (P7f, 7 yrs, WMP3)

However, overall, the concept of managing obesity in the future was a reality for many of the children. Most recognised their positive progress but acknowledged the ongoing effort required for sustained health benefits. This is consistent with the aims of the WMPs, which are to encourage long‐term sustainable lifestyle changes. Children envisioned their future involvement in WMPs, foreseeing continued engagement as a positive aspect:

I will be in high school then, realistically I will probs be still doing this stuff, but this is all good. We are much healthier now as a family, so in a years time, we can all be. (P14m, 10, WMP2)

Despite wishes for instant health transformations, children acknowledged the ongoing journey of self‐improvement, the children were also able to attach reality to the concept of managing obesity over time, identifying how they would still differentiate themselves from children who do not manage their weight:

I'm not sure. Maybe I will be coming to this still; I definitely won't be going to any other clubs after school. I just will never fit in with them sorts. (P18, female, 10 yrs, WMP2)

And

If I could make a wish so that tomorrow I was super healthy and everything was good. I would be cool, like really pretty and have the best clothes. I would laugh a lots, and like, have loads of friends and be able to do loads. …you think?… I would be able to play all the sports games, and I could eat whatever I wanted without being fat, hehe, I would be really thin and, is that ok? But it's not going to happen. My mum won't win the lottery either, but you gotta be in it to win it. (P6f, 11 yrs, WMP2)

## 4 DISCUSSION

This study provided an opportunity to interview several children of different ages and genders, all with shared beliefs, experiences of living with obesity and reflecting on their engagement with a WMP. The qualitative nature of this study allowed new insight into the psychosocial world of children living with obesity and attending a WMP; these insights are progressively recognised as a valuable component in developing the evidence base for public health. The children in this study benefited from attending the WMP and recognised positive changes in their lifestyle behaviours related to dietary improvements, reducing sedentary activity and trying out new PAs. They also acknowledged the long‐term life goal of achieving a healthy weight over time and managing obesity over their life course. These are considered positive outcomes and align with the goals of the intervention, as recommended in clinical guidelines.

However, the children reported challenges with self‐esteem, body image and body dissatisfaction, self‐efficacy, social support, coping strategies, autonomy, motivation and idealistic future expectations. Negative emotions impacted their perception and expectations of current and future weight management. While this study did not evaluate the children for mental health diagnosis. It does, however, highlight those children experienced a range of subclinical psychological challenges, which individually or collectively may have impacted the child's overall well‐being and internalisation and subsequent management of obesity. This research analysis does not suggest that the obesity intervention had a negative impact on the children's psychological well‐being itself; rather, it acknowledges that the children were experiencing and recognised some challenges which should be addressed (but at that time were not). Indeed, while there is a lack of research on psychosocial experiences of living with obesity in children, the research that is available suggests that WMP, like these studies within this research, does not have a detrimental impact on psychological well‐being.^16^, ^30^

The children reported various negative emotions and psychosocial difficulties, which may have contributed to or exacerbated their obesity status and thus impacted their weight management. The children were concerned about being judged by others, particularly peers, the consistent prejudice they have experienced, and how this negatively impacts their overall well‐being. The children experienced weight‐related stigmatisation and societal biases involving bullying and peer teasing, which impacted their psychological well‐being and possibly motivation and/or engagement with the WMP.^44^ Previous research also suggests that experience of stigma contributes to negative effects such as binge eating, social isolation, avoidance of health care services and decreased PA.^45^ Research has shown that weight stigma and internalised weight bias diminish mental and physical health with increasing BMI,^46^, ^47^ which may be the case for the children who participated in this research. Some children described avoidant approaches and coping strategies to manage their negative emotions, including downplaying their obesity status and using humour as a distraction and mask the true extent of the emotional impact of weight stigmatisation. Such approaches have not been explored previously, so further work is needed to understand how to help these children regulate their emotions. Peer bullying interventions that tackle body shaming and weight‐related stigmatisation in children are also warranted.

The children who participated in WMP believed that they had made positive steps towards becoming healthy, trying new foods and activities. These steps made them feel more capable of becoming healthy and increased their self‐esteem and self‐confidence. Consistent with the previous literature,^48^ the strongest evidence was found for improvements in PA, and changing diet was accompanied by enhanced self‐esteem. Despite attending WMPs, some children felt that their psychological well‐being was not directly addressed. While they acknowledged the positive impact of WMP on their lifestyle and confidence in trying new activities, this confidence often did not extend to their daily lives outside the programme.

### 4.1 Clinical implications and future research

The findings from this study have implications for WMPs, in terms of understanding their client's needs (children living with obesity) and how to offer treatment services. There appears to be a gap in the provision regarding supporting these children's psychosocial and emotional well‐being (which may be subclinical mental health). Children living with obesity require support to recognise and manage their feelings and emotions healthily and positively. For example, psychological issues, such as shame (as identified in this study), can be targeted with techniques to build self‐esteem and body acceptance, and children should be supported to develop practical coping skills to manage the effects of stigma and bullying. Obesity services may consider reviewing the behaviour change techniques adopted within the intervention to encourage children to adopt self‐talk, provide stress management sessions, opportunities for social comparison and information about others' approval of the WMP. Families need to be engaged in communication skill development to understand the emotional burden and experiences that children with obesity may experience. Future research should explore parents' (families) perspectives of living with obesity and develop tailored support to resolve conflicts, helping families to recognise their children's psychological and emotional needs and foster positive affect. Services which provide obesity interventions need to skill up staff beyond that of basic behaviour change (e.g., goal setting and motivational interviewing techniques) and should consider employing a psychological practitioner to enhance the programme. Health professionals involved in WMP need to reflect on their implicit and explicit bias and potential stigma that might be transferred to the children living with obesity.^49^ WMP needs to help the children manage experiences of internal bias and external stigma from family, friends, schoolteachers, health professionals or other.^50^ Further research may be helpful to explore the children's perceptions of change, as they journey through a WMP, and how such change impacts their school experiences and the social support around them.

Previous research highlights that 30%–60% of overweight children display symptoms of mental health disorders,^51^, ^52^ while this study did not evaluate the participant's psychological clinical significance, given the range and number of psychological issues highlighted from these participants, we would recommend children with obesity receive a full psychological assessment as part of standardised care. Age and developmentally appropriate psychometric measures can be used to assess psychological well‐being, tailoring interventions to individual needs and demonstrating changes throughout the programme. These measures may assess stress, mindfulness and self‐compassion, emotion regulation, self‐efficacy for healthy food choices,^53^ self‐efficacy for being physically active,^54^ depressive symptoms, quality of life, stigma, internal bias, bullying or resilience. Utilising such measures can help track effective clinical outcomes. Moreover, it may be helpful to assess the attitudes, beliefs, experiences and skills of parents in relation to obesity, weight stigma, teasing and bullying, as well as their techniques to promote behaviour change and lifestyle decisions relating to diet and PA. Ultimately psychological support should be personalised towards the child and family and integrated within the obesity intervention to help the child manage the challenges associated directly with the label, stigma and subsequent management of obesity.

Finally, the children within this study described themselves using negative words such as ‘fat’. Previous literature suggests the terms ‘fat’ and ‘obese’ were viewed to be undesirable, offensive and the least motivating at the point of weight management, stopping children from adopting a healthy lifestyle.^55^, ^56^ Therefore, children should be supported to develop positive body images, and the use of positive language should be promoted. Moreover, for health professionals, person‐first language (i.e., ‘child living with obesity’ as opposed to ‘obese child’) should be adopted throughout the service.^45^, ^57^

### 4.2 Strengths and limitations

As recognised within the obesity literature,^58^ ^,p.368^ there is a need to understand the ‘perspectives and preferences of consumers’ in this context to consult with the service users of the obesity intervention, that being the children and their families. A strength of this study, and its trustworthiness, was that the interviews were conducted with children living with obesity, and the findings within this study may help shape future WMPs. Due to the nature of the interviews, careful consideration of the interviewing techniques was applied so as not to cause any distress to the children, demonstrating a commitment to ethical conduct during data collection, which enhances the credibility of the study. Providing a scrapbook to encourage the children (as suggested by the young people in the PPIE study design process) to think about the interview questions and draw their feelings before the interviews was a novel and successful way to help engage the children and take ownership of the interview content. This is a methodology which would be useful to use in future research given the scrapbooks worked as a tool to aid the children's flow of conversation, the scrapbook use was especially helpful when the topics were sensitive—such as asking the children to talk about their feelings, or how they thought others perceived them, because they were living with obesity.

It is important to highlight that a parent or significant other was present in the room for 10 of the participants during the interviews. While the interviewer thought that the children provided rich and honest information, it may be that the presence of this significant other influenced some of these interviews.

Participants were selected from three independent but similar WMPs; they adopted behaviour change techniques to improve their lifestyles. Behaviour change techniques^34^ employed by the WMPs included self‐monitoring, providing instruction and demonstrating new behaviours while attending the programmes. The WMPs themselves may have already improved the children's quality of life and emotional well‐being before being interviewed, and the children may not have recognised this influence. However, the children in our study revealed various psychosocial challenges, though these were not assessed for clinical significance.

With reference to the dependability of data and analysis, the participants in this study reflected the inclusion criteria for the WMPs and comprised both males and females across the valid age range. Data has not been analysed separately according to gender, though it is possible that males' and females' psychological needs do differ. Two of the programmes were delivered within areas of high deprivation, and one was within an affluent area. The individual socioeconomic characteristics of individual participants were not recorded, and the findings have not made comparisons of data across the WMPs specifically. However, quotes used as evidence within this article have been taken from several participants recruited across all three of the WMPs. Children's experiences of obesity depend on the social context and environment in which they live, and their expectations and experiences of any WMP will depend on the delivery, content and context, which varies across programmes. Nevertheless, to help our readers assess the transferability of the findings to other contexts, we offer a comprehensive description of the methodology and analytical process, with a selection of representative raw data quotes as evidence from across the data set to support our analysis.

## 5 CONCLUSION

This study highlights a range of psychosocial and emotional difficulties that children living with obesity experience and suggests that these remain regardless of their attendance at a WMP. Improving treatment for children living with obesity should address psychosocial factors, including stress management, peer victimisation and handling feedback from others. The novel use of scrapbooks, as suggested by the PPIE as a preinterview tool, was particularly helpful to aid discussion during the interviews and may be a methodological technique to explore in future research on sensitive topics with children.

## AUTHOR CONTRIBUTIONS

**Lisa Newson**: Conceptualisation; investigation; writing—original draft; writing—review and editing; visualisation; methodology; formal analysis; project administration; data curation. **Nicola Sides**: Writing—review and editing; writing—original draft; investigation; formal analysis. **Amineh Rashidi**: Methodology; validation; formal analysis.

## CONFLICTS OF INTEREST STATEMENT

Before this research, Lisa Newson has worked in NHS obesity weight management services. Nicola Sides has previously worked at a UK childhood weight management service. Neither of these interventions was included in this research investigation. The remaining author declares no conflicts of interest.

## ETHICS STATEMENT

Ethical approval was sought from Liverpool John Moores University (PsyRep/290320).

## Data Availability

The raw data supporting this study's findings are available as quotations within the article; examples of text from the scrapbooks have been included where appropriate. Due to the nature of this research, in line with legal and ethical processes, participants did not agree for their full transcripts to be shared publicly, so supporting data beyond the sample data extracts is not feasible.

## References

[1] Mirza NM , Yanovski JA . Prevalence and consequences of pediatric obesity. In: Mirza NM , Yanovski JA , eds. Handbook of Obesity: Epidemology, Etiology, and Physiopathology. Taylor & Francis Ltd.; 2014:55‐74.

[2] Simmonds M , Llewellyn A , Owen CG , Woolacott N . Predicting adult obesity from childhood obesity: a systematic review and meta‐analysis. Obes Rev. 2016;17(2):95‐107. 26696565 10.1111/obr.12334

[3] Ward ZJ , Long MW , Resch SC , Giles CM , Cradock AL , Gortmaker SL . Simulation of growth trajectories of childhood obesity into adulthood. N Engl J Med. 2017;377:2145‐2153. 29171811 10.1056/NEJMoa1703860 PMC9036858

[4] McCullough N , Muldoon O , Dempster M . Self‐perception in overweight and obese. Child Care Health Dev. 2009;35(3):357‐364. 19134011 10.1111/j.1365-2214.2008.00924.x

[5] British Psychological Society . Obesity in the UK: A Psychological Perspective (Obesity Working Group 2011). British Psychological Society; 2011.

[6] British Psychological Society . Psychological Perspectives on Obesity: Addressing Policy, Practice and Research Priorities. The British Psychological Society; 2019.

[7] Curtis P . The experiences of young people with obesity in secondary school: some implications for the healthy school agenda. Health Soc Care Community. 2008;16(4):410‐418. 18328053 10.1111/j.1365-2524.2008.00759.x

[8] Rupp K , McCoy SM . Bullying perpetration and victimization among adolescents with overweight and obesity in a nationally representative sample. Child Obes. 2019;15(5):323‐330. 31062988 10.1089/chi.2018.0233 PMC7364321

[9] Franklin J , Denyer G , Steinbeck KS , Caterson ID , Hill AJ . Obesity and risk of low self‐esteem: a statewide survey of Australian children. Pediatrics. 2006;118(6):2481‐2487. 17142534 10.1542/peds.2006-0511

[10] Mühlig Y , Antel J , Föcker M , Hebebrand J . Are bidirectional associations of obesity and depression already apparent in childhood and adolescence as based on high‐quality studies? A systematic review. Obes Rev. 2016;17(3):235‐249. 26681065 10.1111/obr.12357

[11] Griffiths LJ , Parsons TJ , Hill AJ . Self‐esteem and quality of life in obese children and adolescents: a systematic review. Int J Pediatr Obes. 2010;5(4):282‐304. 20210677 10.3109/17477160903473697

[12] Schwimmer JB . Health‐related quality of life of severely obese children and adolescents. JAMA. 2003;289(14):1813‐1819. 12684360 10.1001/jama.289.14.1813

[13] Mustillo S , Worthman C , Erkanli A , Keeler G , Angold A , Costello EJ . Obesity and psychiatric disorder: developmental trajectories. Pediatrics. 2003;111(4 pt 1):851‐859. 12671123 10.1542/peds.111.4.851

[14] Lobstein T , Baur L , Uauy R . Obesity in children and young people: a crisis in public health. Obes Rev. 2004;5(suppl 1s1):4‐85. 15096099 10.1111/j.1467-789X.2004.00133.x

[15] Puhl RM , Heuer CA . The stigma of obesity: a review and update. Obesity. 2009;17(5):941‐964. 19165161 10.1038/oby.2008.636

[16] Skinner AC , Staiano AE , Armstrong SC , et al. Appraisal of clinical care practices for child obesity treatment. Part I: interventions. Pediatrics. 2023;151(2):e2022060642. 36622110 10.1542/peds.2022-060642

[17] Anderson LN , Ball GDC . Diet, physical activity, and behavioural interventions for the treatment of overweight or obesity in children and adolescents. Paediatr Child Health. 2019;24(6):377‐382. 31528109 10.1093/pch/pxz006 PMC6735582

[18] Mead E , Brown T , Rees K , et al. Diet, physical activity and behavioural interventions for the treatment of overweight or obese children from the age of 6 to 11 years. Cochrane Database Syst Rev. 2017;6(6):CD012651. 28639319 10.1002/14651858.CD012651 PMC6481885

[19] Oude Luttikhuis H , Baur L , Jansen H , et al. Interventions for treating obesity in children. Cochrane Database Syst Rev. 2009;1:CD001872. 10.1002/14651858.CD001872.pub2 19160202

[20] Patalay P , Hardman CA . Comorbidity, codevelopment, and temporal associations between body mass index and internalizing symptoms from early childhood to adolescence. JAMA Psychiatry. 2019;76(7):721‐729. 30892586 10.1001/jamapsychiatry.2019.0169 PMC6583661

[21] Newson L , Abayomi J . Reframing interventions for optimal child nutrition and childhood obesity: the importance of considering psychological factors. Proc Nutr Soc. 2024:1‐31. 10.1017/S0029665124000028 38205619

[22] National Institute for Health and Care Excellence (NICE) . NICE Clinical Guidance 43. Obesity: Guidance on the Prevention, Identification, Assessment and Management of Overweight and Obesity in Adults and Children. National Institute for Health and Care Excellence (NICE); 2006. 22497033

[23] National Institute for Health and Care Excellence (NICE) . Obesity: Working With Local Communities. NICE Public Health Guidance 42. National Institute for Health and Care Excellence (NICE); 2012.

[24] National Institute for Health and Care Excellence (NICE) . Managing Overweight and Obesity Among Children and Young People: Lifestyle Weight Management Services. NICE Public Health Guidance 47. National Institute for Health and Care Excellence (NICE); 2013.

[25] Gatineau M , Dent M . Obesity and Mental Health. National Obesity Observatory; 2011.

[26] NHS Digital . *National Child Measurement Programme, England, 2021/22 School Year*. UK Government; 2022. https://digital.nhs.uk/data-and-information/publications/statistical/national-child-measurement-programme/2021-22-school-year/age

[27] Wijnhoven TMA , van Raaij JMA , Spinelli A , et al. WHO European Childhood Obesity Surveillance Initiative 2008: weight, height and body mass index in 6‐9‐year‐old children. Pediatr Obes. 2013;8(2):79‐97. 23001989 10.1111/j.2047-6310.2012.00090.x

[28] World Health Organisation . WHO European Childhood Obesity Surveillance Initiative (COSI) Report on the Fourth Round of Data Collection, 2015–2017. WHO Regional Office for Europe; 2021.

[29] Ng M , Fleming T , Robinson M , et al. Global, regional, and national prevalence of overweight and obesity in children and adults during 1980‐2013: a systematic analysis for the Global Burden of Disease Study 2013. Lancet. 2014;384(9945):766‐781. 24880830 10.1016/S0140-6736(14)60460-8 PMC4624264

[30] Sides N , Pringle A , Newson L . The lived experience of weight loss maintenance in young people. Health Expect. 2023;27(1):e13955. 10.1111/hex.13955 PMC10768871 39102734

[31] Braun V , Clarke V . Using thematic analysis in psychology. Qual Res Psychol. 2006;3(2):77‐101.

[32] Braun V , Clarke V . Reflecting on reflexive thematic analysis. Qual Res Sport Exercise Health. 2019;11(4):589‐597.

[33] Braun V , Clarke V . What can “thematic analysis” offer health and wellbeing researchers? Int J Qual Stud Health Well‐Being. 2014;9:26152. 25326092 10.3402/qhw.v9.26152 PMC4201665

[34] Abraham C , Michie S . A taxonomy of behavior change techniques used in interventions. Health Psychol. 2008;27(3):379‐387. 18624603 10.1037/0278-6133.27.3.379

[35] Public Health England . Local Authority Health Profiles. Public Health England; 2019.

[36] Elder NC , Miller WL . Reading and evaluating qualitative research studies. J Fam Pract. 1995;41(3):279‐285. 7650507

[37] Ponizovsky‐Bergelson Y , Dayan Y , Wahle N , Roer‐Strier D . A qualitative interview with young children: what encourages or inhibits young children's participation? Int J Qual Methods. 2019;18:160940691984051.

[38] Britten N . Qualitative interviews in healthcare. In: Pope C , Mays N , eds. Qualitative Research in Health Care. 2nd ed. BMJ Books; 1999:11‐19.

[39] Braun V , Clarke V . One size fits all? What counts as quality practice in (reflexive) thematic analysis? Qual Res Psychol. 2020;18:1‐25.

[40] Braun V , Clarke V . Successful Qualitative Research: A Practical Guide for Beginners. Sage; 2013.

[41] QSR International Pty Ltd . NVivo qualitative data analysis software (Version 10) [computer program]. 2014.

[42] Finlay L , Gough B . Reflexivity: A Practical Guide for Researchers in Health and Social Sciences. Blackwell Science; 2003.

[43] Willig C . Introducing Qualitative Research in Psychology. 2nd ed. Open University Press; 2008.

[44] Newson L , Povey R , Casson A , Grogan S . The experiences and understandings of obesity: families' decisions to attend a childhood obesity intervention. Psychol Health. 2013;28(11):1287‐1305. 23758103 10.1080/08870446.2013.803106

[45] Pont SJ , Puhl R , Cook SR , Slusser W . Stigma experienced by children and adolescents with obesity. Pediatrics. 2017;140(6):e20173034. 29158228 10.1542/peds.2017-3034

[46] Gmeiner MS , Warschburger P . Intrapersonal predictors of weight bias internalization among elementary school children: a prospective analysis. BMC Pediatr. 2020;20:408. 32859162 10.1186/s12887-020-02264-w PMC7456014

[47] Lewis K , Fraser C , Manby M . ‘Is it worth it?’ A qualitative study of the beliefs of overweight and obese physically active children. J Phys Activity Health. 2014;11(6):1219‐1224. 10.1123/jpah.2012-0295 23963828

[48] Lubans D , Richards J , Hillman C , et al. Physical activity for cognitive and mental health in youth: a systematic review of mechanisms. Pediatrics. 2016;138(3):e20161642. 27542849 10.1542/peds.2016-1642

[49] Rankin J , Matthews L , Cobley S , et al. Psychological consequences of childhood obesity: psychiatric comorbidity and prevention. Adolesc Health Med Ther. 2016;7:125‐146. 27881930 10.2147/AHMT.S101631 PMC5115694

[50] Palad CJ , Yarlagadda S , Stanford FC . Weight stigma and its impact on paediatric care. Curr Opin Endocrinol Diabetes Obes. 2019;26(1):19‐24. 30516550 10.1097/MED.0000000000000453 PMC6311448

[51] Rao WW , Zong QQ , Zhang JW , et al. Obesity increases the risk of depression in children and adolescents: results from a systematic review and meta‐analysis. J Affect Disord. 2020;267:78‐85. 32063576 10.1016/j.jad.2020.01.154

[52] Van Vlierberghe L , Braet C , Goossens L , Mels S . Psychiatric disorders and symptom severity in referred versus non‐referred overweight children and adolescents. Eur Child Adolesc Psychiatry. 2009;18(3):164‐173. 18807222 10.1007/s00787-008-0717-5

[53] Neumark‐Sztainer D , Wall M , Perry C , Story M . Correlates of fruit and vegetable intake among adolescents. Prev Med. 2003;37(3):198‐208. 12914825 10.1016/s0091-7435(03)00114-2

[54] Sallis JF , Pinski RB , Grossman RM , Patterson TL , Nader PR . The development of self‐efficacy scales for healthrelated diet and exercise behaviors. Health Educ Res. 1988;3(3):283‐292.

[55] Knierim SD , Newcomer S , Castillo A , et al. Latino parents' perceptions of pediatric weight counseling terms. Acad Pediatr. 2018;18(3):342‐353. 28919572 10.1016/j.acap.2017.09.006 PMC5847465

[56] Puhl RM , Peterson JL , Luedicke J . Parental perceptions of weight terminology that providers use with youth. Pediatrics. 2011;128(4):e786‐e793. 21949145 10.1542/peds.2010-3841

[57] Rubino F , Puhl RM , Cummings DE , et al. Joint international consensus statement for ending stigma of obesity. Nat Med. 2020;26(4):485‐497. 32127716 10.1038/s41591-020-0803-x PMC7154011

[58] Smith JD , Fu E , Kobayashi MA . Prevention and management of childhood obesity and its psychological and health comorbidities. Annu Rev Clin Psychol. 2020;16:351‐378. 32097572 10.1146/annurev-clinpsy-100219-060201 PMC7259820

